# Supplementary material for: Pathophysiological Significance of Neutrophilic Transfer RNA-Derived Small RNAs in Asymptomatic Moyamoya Disease
Source: Cells. 2021 May 1;10(5):1086. doi: 10.3390/cells10051086 (PMC8147334; doi:10.3390/cells10051086)

2D Structure	Local AU	Position

```
12                          8mer-1a     42
 5'-gaGAAACCGTATGAATGTGATGA--ATGTGGGa-3' utr
    ||| ||    |:| :|: |  ||  |||||||
 3'-ucCUUAGG---AUUGGCGAU-CUGGUACACCCu-5' tRF
                 16151413      7 6 5 4 3 2
              3' Pairing        Seed
```

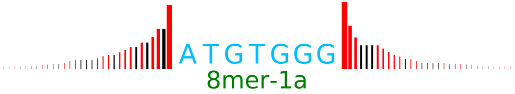

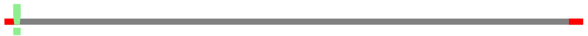

2D Structure	Local AU	Position

```
1166                        7mer-m8     1193
 5'-ttGAAATCT-ATTACTAGTATTTGTGGGt-3' utr
     |||  :||  |::  ||||   : ||||||
 3'-ucCUUAGGAUUGGCGAUCUGGUACACCCu-5' tRF
              16151413    7 6 5 4 3 2
           3' Pairing        Seed
```

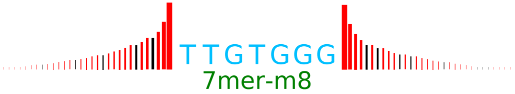

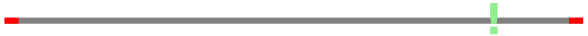

Supplement: Supplementary file 1 [file cells-10-01086-s001.zip › cells-1149161 3rd supplementary materials/Data Supplements/Data Supplement V.zip!/Data supplement 6-mRNAs targets of tsRNAs/tsRNA-Glu-TTC-010/Diagrams/tRF-Glu-TTC-010_vs_ENST00000443905(ZNF506).pdf]
